# Frequency-based haplotype reconstruction from deep sequencing data of bacterial populations

**DOI:** 10.1093/nar/gkv478

**Published:** 2015-05-18

**Authors:** Sergio Pulido-Tamayo, Aminael Sánchez-Rodríguez, Toon Swings, Bram Van den Bergh, Akanksha Dubey, Hans Steenackers, Jan Michiels, Jan Fostier, Kathleen Marchal

**Affiliations:** 1 Department of Information Technology, Ghent University, iMinds, 9050 Gent, Belgium; 2 Department of Microbial and Molecular Systems, Centre of Microbial and Plant Genetics, KU Leuven, Kasteelpark Arenberg 20, 3001 Leuven, Belgium; 3 Department of Plant Biotechnology and Bioinformatics, Ghent University, 9052 Ghent, Belgium; 4 Departamento de Ciencias Naturales, Universidad Técnica Particular de Loja, San Cayetano Alto S/N, EC1101608 Loja, Ecuador

## Abstract

Clonal populations accumulate mutations over time, resulting in different haplotypes. Deep sequencing of such a population in principle provides information to reconstruct these haplotypes and the frequency at which the haplotypes occur. However, this reconstruction is technically not trivial, especially not in clonal systems with a relatively low mutation frequency. The low number of segregating sites in those systems adds ambiguity to the haplotype phasing and thus obviates the reconstruction of genome-wide haplotypes based on sequence overlap information.

Therefore, we present EVORhA, a haplotype reconstruction method that complements phasing information in the non-empty read overlap with the frequency estimations of inferred local haplotypes. As was shown with simulated data, as soon as read lengths and/or mutation rates become restrictive for state-of-the-art methods, the use of this additional frequency information allows EVORhA to still reliably reconstruct genome-wide haplotypes. On real data, we show the applicability of the method in reconstructing the population composition of evolved bacterial populations and in decomposing mixed bacterial infections from clinical samples.

## INTRODUCTION

The genetic heterogeneity of clonal populations is key to their adaptive behavior. Environment-specific genes, subject to relaxed selection in a non-inducing environment, build up cryptic variation, that enhances the adaptive potential (1,2). Even when starting evolution from a single clone (haplotype) under severe selection pressure, the combination of mutation rate and population size appears to be sufficiently high to build up genetic variation in the population (3,4), resulting in a mixture of closely related haplotypes (or quasispecies). As a result, a population is not genetically uniform most of the time (5). Although single cell sequencing would be ideal to determine the composition of such a heterogeneous population, it is still technically very difficult and cost-inefficient (6–8). However, deep sequencing a clonal population in its entirety, referred to as pooled or metagenomic sequencing (9) inherently contains information to determine the haplotypic variation of the population, i.e. the identity of the occurring haplotypes and their frequencies.

However, resolving haplotypes from deep sequencing data of clonal populations, also referred to as haplotype reconstruction or quasi-species assembly is technically not trivial and methods to do so are still lacking for most clonal species, other than viruses.

At first because the problem of error correction is confounded with the haplotype reconstruction itself (10) and therefore error correction and haplotype reconstruction should ideally be performed simultaneously. The reconstruction problem itself is non-trivial either. For this reconstruction step all current haplotype reconstruction methods rely on the presence of a sufficient number of segregating sites and relatively long reads to allow phasing the segregating polymorphic sites into unique haplotypes using either single end (11–16) or paired end read information (17). This strategy implies that most reads should contain segregating sites and that a sufficient amount of overlap between reads is available to resolve the reconstruction problem. As a result, current methods are restricted to haplotype reconstruction from relatively long-read population sequencing (mainly Roche 454) of clonal organisms with a high mutation frequency (18), such as viruses: a high mutation frequency guarantees a large number of segregating sites and long-read based sequencing allows for a large degree of overlap between the reads.

However, for most clonal populations the number of observed segregating sites is much lower than what is observed in viral populations. In a bacterial setting, for instance, haplotypes consist of millions of base pairs corresponding to the size of a bacterial genome, but populations typically contain less than a few hundreds of mutations even in the presence of a mutator phenotype (e.g. bacterial populations originating from a mutator phenotype accumulate after 300 generations approximately 1000 mutations). This relatively low mutation frequency implies an average distance between segregating sites that is in the order of kilobases, which is a lot larger than the maximal read length for Illumina and Roche 454 technologies. Due to the lack of segregating sites, phasing becomes extremely difficult. As a result, state-of-the-art viral haplotype reconstruction methods cannot infer haplotypes from bacterial population samples.

With EVORhA (Evolutionary Reconstruction of Haplotypes) we propose to the best of our knowledge the first bacterial haplotype reconstruction method. EVORhA combines local haplotype inference with error correction and uses a probabilistic approach for the genome-wide reconstruction. Key to the method is the use of the inferred frequency ratios of the contributing haplotypes to improve the extension of local haplotypes into genome-wide ones, particularly in those cases where the non-empty overlap between reads does not allow for a non-ambiguous phasing or where partially reconstructed regions have no sequence overlap at all. Because of this key step EVORhA is applicable to the analysis of pooled sequence data obtained from populations of clonal organisms with a low mutation frequency and/or to data obtained with a short-read based technology. We demonstrated the performance of EVORhA under different settings on simulated data. In addition, we showed its ability to reconstruct genome-wide haplotypes in a real setting by analyzing data obtained from a mixed bacterial infection and from pooled sequence samples of an evolving bacterial population. The implementation can be downloaded from http://bioinformatics.intec.ugent.be/kmarchal/EVORhA/. The source code is available upon request.

## MATERIALS AND METHODS

### EVORhA

**Figure 1.**
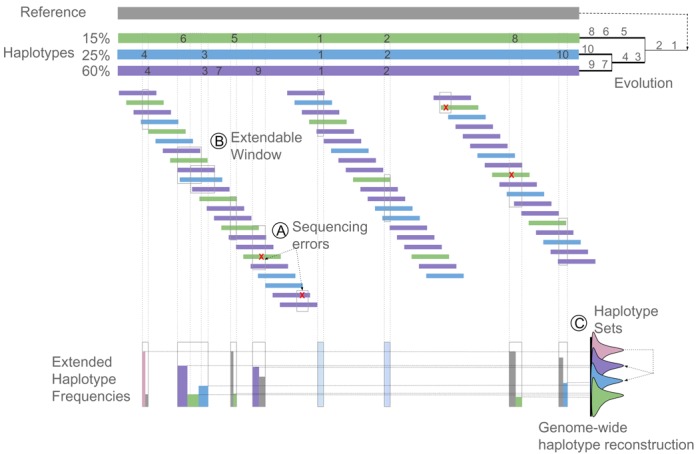
Method Overview. A clonal community where three descendant haplotypes evolved from a reference genome is depicted. The haplotypes are present at respectively 60%, 25% and 15% in the population. A total of 10 different mutations accumulated in the evolving population. Mutations ‘1’ and ‘2’ were acquired by the last common ancestor of extant haplotypes and are therefore shared by all haplotypes in the population. Mutations ‘3’ and ‘4’ were acquired before the origin of the *blue* and *purple* haplotypes, whereas the remaining mutations are unique to one of the haplotypes. Small colored horizontal bars represent the reads obtained by deep sequencing the aforementioned population. The coloring of the reads corresponds to the colors of the haplotypes from which they originated. Reads are mapped to the ancestral reference genome. Step 1: Local haplotype reconstruction and window extension. (**A**) Haplotype templates and their frequencies are first inferred per window. Windows are represented by gray rectangles. A window is defined as a genomic region that is covered by a sufficient number of reads and that contains a set of one or more consecutive tentative polymorphic sites. Tentative polymorphisms can refer to both sequencing errors (red crosses) and true polymorphisms. Per window, accepted template windows will be defined by performing a local haplotype reconstruction simultaneously with the error correction (see Supplementary Figure S1). (**B**) Template haplotypes are extended over flanking windows with overlapping polymorphic sites based on the consistency in the polymorphisms present in the non-empty read overlap of these flanking windows and in the frequencies of the extended template haplotypes. Two windows for which the ‘window extension’ will be performed are indicated by gray rectangles. In the example the template haplotypes within these flanking windows will be merged into 3 extended haplotypes (the *purple*, *green* and *blue* one, see Supplementary Figure S2). (**C**) Step 2: Genome-wide haplotype reconstruction. Extended haplotypes from the different concatenated windows will be merged into genome-wide haplotypes based on the frequency information. To this end, we use a mixture model approach in which extended haplotypes occurring at similar frequencies in the population, referred to as haplotype sets are represented by a different mixture component in the model (distributions drawn at the right of the picture). Haplotype sets containing polymorphisms unique for the haplotype will occur at a lower frequency than haplotype sets that contain polymorphisms shared by several genome-wide haplotypes (indicated in pink, see also Supplementary Figure S3). The genome-wide haplotype reconstruction searches for combinations of haplotype sets that provide the best explanation for the observed frequencies of all haplotype sets.

Our method uses a two-step procedure: the first step reconstructs haplotypes at the local level and joins locally reconstructed haplotypes into so-called extended haplotypes based on information contained within the read overlap. The second step assigns these extended haplotypes to haplotype sets by using a mixture model of Gaussian distributions that describes for each set the frequency at which the haplotypes assigned to the set are observed. These haplotype sets are eventually joined into genome-wide haplotypes, following a procedure that explicitly assumes that the different haplotypes in the population have evolved from a common ancestor by clonal reproduction. The procedure is outlined in Figure 1.

#### Step 1: Local haplotype reconstruction and window extension

##### Window definition

A window is defined as a genomic region that is covered by a sufficient number of reads and that contains a set of one or more consecutive tentative polymorphic sites. As at this point, variation observed at these sites can refer to both sequencing errors and true polymorphisms, we refer to them as ‘tentative’. All windows that contain a unique combination of consecutive (tentative) polymorphic sites are enumerated, with the restriction that windows should be shorter than a pre-specified maximal window length (60% of the read length by default) and that windows should be entirely covered by a certain minimum number of reads (30 reads by default). Regions that are not covered by the minimal number of reads will be ignored. Both window-defining parameters could be adjusted if necessary.

##### Inferring template haplotypes and their frequencies per window

A list of possible template haplotypes is generated per window (see Supplementary Figure S1). A template haplotype is defined as a unique combination of one or more consecutive (tentative) polymorphisms observed in at least one of the reads that fully covers the window. For each template haplotype ***h*** = {*h*_1_, *h*_2_, …} found in window *W* a corresponding ‘support’ $\tau = \left\{ {\tau _1 ,\tau _2 , \ldots } \right\}$ is based on all reads that are consistent with that template haplotype. First, we consider only the reads that fully overlap with the window and calculate a *base support* $\tau _i^0$ for template haplotype *h_i_* as follows:

\begin{equation*} \tau _i^0 = \mathop \sum \limits_{r\, \in \,F_i } w\left( r \right)\end{equation*}

where *F_i_* denotes the set of reads that fully overlap with the window and that are consistent with template haplotype *h_i_* and where *w*(*r*) is given by

\begin{equation*} w\left( r \right) = \mathop {\min }\limits_{j = 1,l} P\left( {r\left[ j \right]} \right)\end{equation*}

with $P\left( {r[j]} \right)$ the base call accuracy at tentative polymorphic site *j* of read *r* containing *l* tentative polymorphisms. It is related to the Phred quality score $Q(r[j])$ as follows:

\begin{equation*} P\left( {r\left[ j \right]} \right) = 1 - 10^{\frac{{ - Q\left( {r\left[ j \right]} \right)}}{{10}}} \end{equation*}

We choose w(r) to depend on min $P(r[j])$, assuming that the contribution of the read to the support depends on its lowest quality polymorphism. This allows to have a scoring independent of the window length.

Additional support $\tau _i^1$ for template haplotype *h_i_* is derived from reads that only partially overlap with window *W*.

\begin{equation*} \tau _i^1 = \mathop \sum \limits_{r \in P_i } \frac{{\tau _i^0 }}{{\mathop \sum \nolimits_{j|r \in P_j } \tau _j^0 }}w\left( r \right)\end{equation*}

Where *P_i_* denotes the set of reads that partially overlap with the window and that are consistent with template haplotype *h_i_*. Note that partially overlapping reads can be consistent with multiple template haplotypes. The fraction within the summation therefore denotes that a given read gives a support to the haplotype it is compatible with, weighted proportionally to the base support of that haplotype. The total support of a template haplotype $\tau _i$ is then given by

\begin{equation*} \tau _i = \tau _i^0 + \tau _i^1 \end{equation*}

The template haplotypes ***h*** might contain errors, i.e. tentative polymorphisms that arose due to sequencing errors. To prune templates in the windows, we retain per window only the template haplotypes with support $\tau _i$ greater than the template haplotype threshold. The threshold for the support is different for different templates and is determined by the following equation:

\begin{equation*} \tau _{{\rm threshold}} = - 11.86 + 4.24\ln \left( f \right) + E\left( c \right)\end{equation*}

\begin{equation*} E\left( c \right) = \left\{ {\begin{array}{*{20}c} {0\,if\,InterGenic{\mathop{Region}\nolimits}} \\ {0\,\,{if}\,\,{BLOSUM} \ge 0} \\ { - 1 \times {BLOSUM}\,\,{if}\,\,{BLOSUM} < 0} \\ \end{array}} \right.\end{equation*}

Where *f* represents the average fold coverage in the considered window to which a polymorphism belongs and *c* the codon in the same window with the lowest BLOSUM score. The BLOSUM matrix used is obtained by comparing already calculated matrices against the analyzed data. In most cases, the most similar matrix is the BLOSUM100. The equation describes that the minimal threshold on the support for accepting template haplotypes will increase with the coverage of the window and with the severity of the amino acid changes induced by the polymorphisms, i.e. template haplotypes with a higher coverage and more unlikely amino acid changes need more support to be retained. The parameters in this formula, being the contribution of the window coverage and the codon changes, were determined by maximizing the accuracy of reconstructing true haplotypes in a simulated setting (see simulated data).

A haplotype with a support below the threshold is assumed to be a ‘false positive’ and will no longer be considered as a possible template. Reads that contributed to the support of a rejected template will be reassigned to the accepted template haplotype that is evolutionary most related to it (using the BLOSUM matrix mentioned above). Note that sequence error correction is performed simultaneously with haplotype reconstruction: rather than filtering upfront tentative polymorphisms that occur infrequently (i.e. standard error correction), polymorphisms are filtered when they belong to template haplotypes with insufficient support. This prevents the deletion of infrequently observed polymorphisms when they belong to a template haplotype with sufficient support.

##### Window extension: concatenating windows that share polymorphisms

Here, we start with a set of windows and their respective accepted template haplotypes. Some windows will share polymorphic sites and will be extended (see Supplementary Figure S2). The extension is performed window by window, starting from a so called seed window which corresponds to the window with the largest number of ‘accepted’ template haplotypes, the largest number of polymorphic sites and the highest coverage. If no window meets all criteria simultaneously, we select the seed window by prioritizing first on the number of template haplotypes, then on the number of polymorphic sites and, lastly, on coverage. The goal of the extension is to find the best combination of haplotypes from the first and second window that can be concatenated to generate an extended haplotype, where ‘best’ is defined in terms of matching frequencies and shared polymorphisms.

The extension, which is conceptually very similar to what is referred to as the ‘global reconstruction’ in graph-based haplotype reconstruction approaches (11–13,16), is performed as follows: for each pair of overlapping windows $W = \left\{ {W_a ,W_b } \right\}$, a set of *groups G* is declared where one group *g_i_* is defined per unique combination of consecutive polymorphisms in the overlap of both windows. The template haplotypes in the windows are then assigned to those groups. Note that a specific template haplotype can only be assigned to a single group; however, a certain group can contain multiple template haplotypes. We can now distinguish three cases:

1. If a group contains a single template haplotype in one window and at least one template haplotype in the other window, the extension is straightforward and the extended haplotypes consist of the concatenation of the sequences of the template haplotypes in both windows.
2. If a group contains multiple template haplotypes in both windows, the extension is ambiguous. In that case, first, it is assumed that the number of extended haplotypes equals the maximum number of template haplotypes present in either window. The assignment of template haplotypes to the extended haplotypes and the frequencies of the extended haplotypes $\theta = \left\{ {\theta _1 ,\theta _2 , \ldots } \right\}$ are determined using an expectation maximization algorithm. First, the frequencies *θ* are initialized randomly. During the expectation step, template haplotypes in both windows are assigned to the extended haplotypes such that the observed frequencies *f* of the template haplotypes best match the frequencies *θ*. In case a template haplotype can be assigned to multiple extended haplotypes, the frequency of the template haplotype is split according to the frequencies *θ* of the extended haplotypes to which it was assigned. All possible combinations of assignments are exhaustively enumerated and the one that maximizes the log-likelihood according to the Poisson distribution is selected:

\begin{equation*} L = \mathop \sum \limits_{x \in X} \left[ {\log \frac{{\lambda _{x,a} ^{k_{x,a} } }}{{k_{x,a} !}}e^{ - \lambda _{x,a} } + \log \frac{{\lambda _{x,b} ^{k_{x,b} } }}{{k_{x,b} !}}e^{ - \lambda _{x,b} } } \right]\end{equation*}

where $\lambda _{x,a} = c_a \theta _x$ and $\lambda _{x,b}$ = $c_b \theta _x$ denote the expected number of reads matching extended haplotype *x* in windows *a* and *b*, respectively. Here, *c_a_* and *c_b_* are the coverages in windows *a* and *b*, respectively and *θ_x_* is the frequency of extended haplotype *x*. Similarly, $k_{x,a} = c_a f_x$ and $k_{x,b}$ = $c_b f_x$ denote the observed number of reads matching extended haplotype *x* in windows *a* and *b*, respectively, with *f_x_* being the observed frequency of the template haplotypes.

The maximization step then computes the new frequencies *θ* for extended haplotypes by computing the average frequencies of the contributing template haplotypes.

This process is repeated until the likelihood difference between consecutive iterations becomes sufficiently small or until a maximum number of iterations has been reached. We perform multiple starts with random initial frequencies to avoid local maxima.

If a certain group only contains template haplotypes from one window (not both), the template haplotypes in the group are moved to a different group that contains the haplotypes for which the evolutionary distance (BLOSUM) to the haplotypes under consideration is the smallest. This situation can occur with low frequency haplotypes where reads derived from these haplotype might not be available for all windows. After reassigning the haplotypes to another the procedure is as described in (1) and (2).

The concatenated window containing the extended haplotypes is subsequently used as a seed to concatenate the next set of flanking windows. If no more flanking windows exist for the current concatenated window, a new initial window is defined. The procedure continues until all windows that display overlapping polymorphisms have been concatenated.

The window extension thus produces a set of extended windows and their respective extended haplotypes. An extended window by definition does not share any polymorphic sites with other windows and cannot be further extended (phased) by analyzing read overlap.

#### Step 2: Genome-wide haplotype reconstruction

Extended haplotypes are those that can no further be concatenated, because they do not longer contain polymorphisms in their read overlap. This occurs when either the read overlap between two extended haplotypes is empty or non-informative. The latter situation arises in case of low mutation frequency when the genomic distances between segregating sites are usually larger than the median read length. To compensate for this lack of information, we will use the frequency information of each of these extended haplotypes, to infer a possible genome-wide haplotype. To combine extended haplotypes into genome-wide haplotypes, we first perform a frequency analysis by grouping together extended haplotypes that occur at similar frequency (referred to as haplotype sets) and subsequently use a power set approach to search for a final genome-wide haplotype that can explain the frequencies of the observed sets of ‘extended haplotypes’.

##### Frequency analysis

Let ***R*** = {*R*_1_, *R*_2_, …, *R_n_*} denote the number of reads that correspond to the extended haplotypes ***h*** = {*h*_1_, *h*_2_, …, *h_n_*}, observed in a concatenated window. ***R*** then follows a multinomial distribution:

\begin{equation*} {\boldsymbol{R}}\sim{\rm Multi}\left( {C,{\boldsymbol{P}}} \right)\end{equation*}

where *C* denotes the number of reads observed in the concatenated window and ***P*** = {*P*_1_, *P*_2_, …, *P_n_*} denotes the true frequencies of the haplotypes. If the window coverage is sufficiently high, the multinomial distribution ***R*** can be approximated by *n* normal distributions:

\begin{equation*} R_i = CX_i \sim {\rm N}\left( {CP_i ,CP_i \left( {1 - P_i } \right)} \right)\end{equation*}

where ***X*** = {*X*_1_, *X*_2_, …, *X_n_*} are the observed frequencies of the extended haplotypes in the concatenated window. It then follows that:

\begin{equation*} X_i \sim {\rm N}\left( {P_i ,\frac{{P_i \left( {1 - P_i } \right)}}{C}} \right)\end{equation*}

Given that the extended haplotypes and the frequencies ***X*** at which they are observed, should be consistent over at least several windows, the frequencies at which extended haplotypes are observed in each of the windows can be assumed to be generated by a mixture model of Gaussian distributions (see Supplementary Figure S3). If polymorphisms occurring at the same site are shared by different haplotypes, they will occur at a frequency (or Gaussian) different than polymorphisms that are unique to a single haplotype.

This mixture model is inferred as follows: the method starts by assigning one Gaussian distribution to each of the extended haplotypes observed in an initially selected concatenated window (using the same initialization criteria defined above i.e. the concatenated window with the largest number of ‘accepted’ template haplotypes, the largest number of polymorphisms and the highest coverage is selected as a seed. If no window meets all criteria simultaneously, we select the seed prioritizing first on the number of template haplotypes, then on the number of polymorphisms and, lastly, on coverage). Subsequently we assign per concatenated window each extended haplotype to the Gaussian that currently best explains its observed frequency provided the mean of this Gaussian is located less than one standard deviation from the observed haplotype frequency. If the mean of the best explaining distribution is more than one standard deviation away from the observed frequency of the given haplotype, we create a new Gaussian in the mixture model and we continue until all haplotypes have been assigned to a Gaussian distribution that is less than one standard deviation away from the observed frequency of the given haplotype. The resulting model is referred to as the full mixture model.

As this is a very relaxed way of extending the mixture, we include a final Bregman hierarchical clustering step (19) to reduce the model complexity and find an optimal mixture model for which the difference with the full mixture model in fitting the observed frequencies of the haplotypes is less than 1%. By considering the frequencies of all extended haplotypes in all extended windows, the frequency analysis results in a mixture model of Gaussian distributions that groups all extended haplotypes, occurring at a similar frequency in haplotype sets.

##### Inferring the final genome-wide haplotype

Each inferred distribution in the mixture model corresponds to a set of extended haplotypes that are likely to co-occur in one or more genome-wide haplotypes (referred to as a haplotype set). At this moment each true haplotype can still be characterized by several Gaussians from the mixture (see Supplementary Figure S3). This is because haplotype sets containing polymorphisms unique for the haplotype will occur at a lower frequency than haplotype sets that contain polymorphisms shared by several genome-wide haplotypes.

To join haplotype sets that can safely be assumed to belong to the same haplotype we use the following approach: a distance matrix *D* is calculated between all pairs *i,j* of haplotype sets, where $D_{i,j} = \left| {P_i \mathop \cup \nolimits^ P_j } \right| - \left| {P_i \mathop \cap \nolimits^ P_j } \right|$ and *P_i_*, *P_j_* are the polymorphisms composing each haplotype set, respectively. Obviously, different polymorphisms at the same polymorphic site are considered as different objects in the haplotype sets.

Subsequently, for each haplotype set *h* from the full list of haplotype sets ${\cal H}$ (always starting with the haplotype set with the highest observed frequency) we construct a subset ${\cal H}_h = \left\{ {g \in {\cal H}|\mu _g < \mu _h \wedge D_{h,g} = 0} \right\}$ where *μ_g_* and *μ_h_* are the means of the Gaussian distributions representing haplotypes sets *g* and *h*, respectively. Then, for the power set $P\left( {{\cal H}_h } \right)$ we construct $P\prime \left( {{\cal H}_h } \right) = \{ {\rm \omega } \in P\left( {{\cal H}_h } \right)|f\left( {\rm \omega } \right) < 2 \times \sigma _h \}$ where *σ_h_* is the standard deviation of the Gaussian distribution representing haplotype set *h* (i.e. those subsets where the sum of the frequencies of the haplotype sets is in the 95% confidence interval of the Gaussian distribution of *h*):

\begin{equation*} \varpi = \mathop {{\rm argmin}}\limits_{{\rm \omega } \in P' \left( {{\cal H}_h } \right)} f\left( {\rm \omega } \right)\end{equation*}

\begin{equation*} f\left( {\rm \omega } \right) = \left[ {\mu _g - \mathop \sum \limits_{{\rm i} \in {\rm \omega }} \mu _i } \right]\end{equation*}

If *ϖ* exists, we conclude that haplotype set *h* contains a set of polymorphisms shared by all haplotypes in *ϖ* and therefore will no longer be considered as an individual haplotype. Therefore, we remove *h* from the list of haplotypes and add the polymorphisms in *h* to all haplotype sets in *ϖ*. This final step in the analysis results in the reconstructed genome-wide haplotypes, their inferred frequencies and polymorphisms.

The following running parameters are used for EVORhA by default:

The parameters of the method are those that define valid windows, being the ‘maximal window length’ and the ‘minimal read coverage’. For the minimal read coverage a default value of 30 reads was chosen. This, to guarantee a sufficient number of reads in each window so that the distribution of the reads corresponding to template haplotypes in the window can be approximated by a multinomial, and therefore can be modeled as a mixture model of Gaussian distributions, such as described in step 2. The default of the maximum window length was chosen at 60% of the read length so that the template haplotypes and their *base support*, both derived from reads that fully overlap with the window, are representative for the true haplotypes present in the window.

### Simulation experiments

#### Performance assessment of EVORhA

To test EVORhA, a first set of population sequence data was generated using the sequence of *Salmonella Typhimurium* 14028S (Accession number CP001362) as the ancestral reference strain. In the simulated populations, the number of polymorphisms varied between 100, 1000 and 2500, the number of haplotypes varied from 2 to 7 and their frequencies were set randomly. For each simulated population a random phylogeny was constructed prior to assigning polymorphisms to haplotypes. Polymorphisms were added at a random branch of the phylogeny and propagated to all haplotypes descending from that branch, ensuring the simulation of evolutionary related haplotypes (assuming that the different haplotypes in the population have developed by clonal reproduction from a common ancestor). For each of these populations we simulated reads at the polymorphic sites for different sequence coverages (ranging between 50, 200 and 500) and using a sequencing error probability of 1%. Per parameter combination (number of haplotypes, number of mutations and coverage), 100 data sets were generated.

The degree to which an inferred haplotype could correctly be reconstructed was assessed by a ‘reliability score’, which is the proportion of shared polymorphisms between the reconstructed haplotype and its most similar true counterpart i.e. the simulated haplotype.

\begin{equation*} Reliability = \frac{{\left| {P_h \mathop \cap P_s } \right|}}{{\left| {P_h } \right| + \left| {P_s } \right|}}\end{equation*}

where *P_h_* and *P_s_* are the polymorphisms present in the reconstructed haplotype and its most similar true counterpart, respectively.

To test the extent to which the true frequencies of the reconstructed haplotypes could be inferred, we used the mean absolute error (MAE) between the true frequencies at which a haplotypes occurred (*y_i_*) in the simulated population and the estimated frequencies of the matching reconstructed haplotypes ($\hat y_i$) divided by the number of true haplotypes (*N*).

\begin{equation*} MAE = \frac{1}{N}\sum {(|y_i - \hat y_i |)} \end{equation*}

#### Comparison with state-of-the-art haplotype reconstruction methods

To perform a comparison with ShoRAH (11), QuasiRecomb (16) and Predicthaplo (15) we simulated a second set, this time consisting of raw population sequence data (as we need an alignment file as input for each of the respective algorithms we compared with). Raw data sets were generated using GemSIM v1.6 (20), derived from a single gene of 2562 bp long, flanked on both sides by 700 bp regions. The number of haplotypes ranged between 2 and 4. The number of polymorphisms in the simulation was varied between 7, 10, 20 and 50. The same phylogenetic approach mentioned above was used to generate evolutionary related haplotypes. Raw data mimicked 100 and 700 bp reads, produced under respectively an Illumina and a Roche 454 error model provided by the simulator. The coverage varied between 50, 100, 200 and 500. One hundred (100) data sets were generated for each combination of parameters. Note that we focused on simulating one gene rather than a full bacterial genome in order to design a set up optimized for the state-of-the-art methods we intended to compare with (as these cannot handle a genome-wide haplotype reconstruction).

The latest version of ShoRAH was downloaded from: http://www.bsse.ethz.ch/cbg/software/shorah. ShoRAH was run according to the authors’ recommendations with a window size that is about one third of the read length, i.e. with a window of 30 bp for simulations with a read length of 100 bp reads and of 252 bp for simulations with read lengths of 700 bp. The latest version of PredictHaplo was obtained from: http://bmda.cs.unibas.ch/HivHaploTyper/. In our hands, most of the simulated data sets could not be processed with PredictHaplo, preventing us from comparing its performance with that of the other methods. The latest version of QuasiRecomb was obtained from: http://www.silva.bsse.ethz.ch/cbg/software/quasirecomb. The method was run with flags ‘noRecomb’, ‘conservative’ and ‘unpaired’ as recommended by the authors for a comparable setting. All tools were run on the same simulated data sets.

### Haplotype reconstruction to infer evolutionary trajectories

The data used for haplotype reconstruction during bacterial evolution were generated as follows: *Escherichia coli* SX4 was grown under selective pressure (high concentration of ethanol) in a serial transfer experiment in which the concentration of ethanol was gradually increased over time (0.5% each time) as soon as the population resumed growth under a current selection pressure. At three consecutive time points, population samples and individual clones, selected from these sampled populations were subjected to Illumina sequencing HiSeq2000 (using 100 bp paired end read mode, with a coverage of approximately 200-fold for the population samples). Sampling and DNA isolation were done according to standard procedures (Qiagen® Blood & Tissue kit). Sequences are stored under BioProject PRJNA262000. Read mapping of both the sequenced pooled samples and individual clones was performed with Burrows–Wheeler Aligner BWA-MEM using the sequence of the original unevolved ancestral clone as a reference. For the sampled clones variants were called after alignment using SAMtools (21) with default parameters. As a control we confirmed that the called variants were also obtained using the CLC Bio pipeline (http://www.clcbio.com) with default parameters.

The true haplotypes of the individual clones sampled from the pool were compared with the haplotypes reconstructed from the pooled data. To this end we calculated the ratio of the number of polymorphisms shared between an individual clone and its best matching reconstructed haplotype versus their total number of polymorphisms. Polymorphisms that reached fixation in the population were not taken into account as they are present in all haplotypes and therefore not informative.

The phylogenetic relations between the haplotypes reconstructed at different time points was inferred using a Levenshtein distance measure between a haplotype observed at a current time point and the ones observed at the closest preceding time point, hereby assuming that the closest haplotype in the preceding time point is the evolutionary ancestor of the haplotype observed at the current time point.

### Haplotype reconstruction to identify mixed infections

Publicly available read mapping data of in-vitro mixed infections were obtained from Eyre et al. (22). They generated 36 mixed infections by pairwisely combining the DNA obtained from different clones in three different proportions—50%/50%, 70%/30% and 90%/10%. For each proportion 12 mixed infections were generated, each consisting of different pairwise combinations of clones. The pools of the *in vitro* generated mixed infections were subjected to Illumina sequencing at 150-fold coverage. We performed a genome-wide reconstruction using all variant loci detected in the population sequencing data of the mixed infection and, as an alternative, we also performed a reconstruction by using a preselected set of 151 polymorphic sites present in 3 different genes as outlined in Eyre et al. (22). For both reconstructions the root-mean-square error (RMSE) was calculated to assess the correctness of predicting the correct haplotype frequencies in the mixed infection. We use the RMSE rather than the above mentioned MAE to be consistent with the original paper of Eyre et al. (22). The reliability of the reconstruction was assessed as described in section ‘performance assessment’ by comparing the polymorphisms present in the reconstructed haplotypes with the polymorphisms observed in the sequences of the single clones.

## RESULTS

Our method consist of two steps: a first step comprising a local haplotype reconstruction followed by a window extension, in which haplotypes are defined at the local level, sequencing errors are removed and overlapping regions sharing polymorphisms are extended into longer haplotypes; a second genome-wide reconstruction, during which the final haplotypes and their relative frequencies are inferred by using the frequency observations of the extended haplotypes.

Based on concepts developed in the context of viral haplotype reconstruction (11,12,15,16), the first step of our method performs the error correction simultaneously with the haplotype inference on a local scale (Figure 1A and B). The local scale is defined by a set of consecutive polymorphic sites that map on a single genomic region of the reference genome and that are ‘covered’ by a sufficient number of reads (referred to as a window). The simultaneous estimation of the sequence errors with the local haplotype inference is based on a method that iterates between assigning reads to haplotypes and using these read-to-haplotype assignments to infer the probabilities that either the observed reads were the result of random sequence error or originated through mutations in the ancestral genome. We used information contained in BLOSUM substitution matrices to lower the allowance of mutations in coding regions, occurring rarely in nature. This local reconstruction step results per genomic window in haplotypes and their observed frequencies in the pooled sample (referred to as local haplotypes, consistent with the literature (10)). Subsequently, these local haplotypes are extended using a heuristic approach that takes into account both phasing information in the non-empty read overlap between flanking windows, and also the inferred local haplotype frequencies. Because of the sparse number of expected segregating sites between the individuals in the population, the haplotype extension step results in slightly larger contigs, but rarely covers more than a few hundred base pairs.

Therefore, in the second step, referred to as the genome-wide haplotype reconstruction, extended haplotypes are joined into genome-wide haplotypes that ideally cover a full haplotype in the population (Figure 1C). This step is entirely dependent on the frequency information of the extended haplotypes: sets of extended haplotypes that occur at a similar frequency in the population and that do not show any inconsistencies in their polymorphisms (i.e. do not have a different mutation at exactly the same genomic position) are assumed to belong to the same genome-wide haplotypes. This genome-wide haplotype reconstruction step is solved by first estimating sets of locally extended haplotypes that occur at a similar frequency and subsequently searching for the set of genome-wide haplotypes and their frequencies that best explain the observed frequencies of the extended haplotypes. This latter step assumes that the haplotypes in the population have developed by clonal reproduction from a common ancestor and therefore haplotype sets that are shared by at least two genome-wide haplotypes should occur at a frequency in the population that approximates the sum of the frequencies of each of the individual genome-wide haplotypes containing the shared haplotype set.

### Performance of EVORhA on simulated data

To test the performance of EVORhA in reconstructing haplotypes, whole genome sequencing data sets were simulated for clonal populations, differing from each other in the number of haplotypes (ranging from 2–7), the frequencies at which the haplotypes occur in each of these populations, the number of polymorphisms (ranging between 100, 1000 and 2500) and the sequencing coverage (ranging between 50-, 200- and 500-fold). For each simulation setting, 100 simulations were performed.

**Figure 2.**
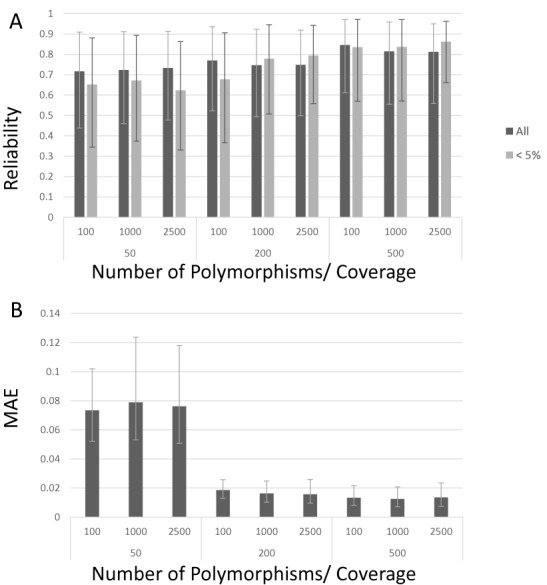
Reliability of haplotype reconstruction by EVORhA on simulated data. The X-axis displays the different combinations of coverage (respectively 50, 200, 500) and number of polymorphisms in the population (respectively 100, 1000, 2500) used for each simulated set up, hereby collapsing the results obtained for simulations with a different number of haplotypes (for the uncollapsed results see Supplementary Figure S4). The degree to which the simulated haplotypes was correctly reconstructed was assessed by the reliability. The correctness of the frequency estimation of the reconstructed haplotypes was assessed by the MAE. (**A**) Average reliability of the haplotype reconstruction, derived by either considering the results of all reconstructed haplotypes (dark bars) or only the results obtained for haplotypes that occur in the population at a frequency below 5% (light bars). Y-axis: average reliability; values are obtained by averaging the reliability scores of the considered haplotypes resulting from simulations obtained with the same coverage and same number of polymorphisms, irrespective of the number of haplotypes (so showing the average reliability of haplotypes obtained from 500 simulations). Error bars indicate the 90% confidence interval of the reconstruction. (**B**) Y-axis: MAE of the frequency estimation of all haplotypes resulting from simulations obtained with the same coverage and same number of polymorphisms, irrespective of the number of the haplotypes (see panel (A)). Error bars indicate the MAE 90% confidence interval.

To assess the reliability of the reconstruction, we compared the reconstructed haplotypes with the simulated ones. At the same time we assessed the ability of the reconstruction to correctly estimate the true frequencies at which the haplotypes occurred in the population. As expected, the reliability of the reconstruction increases with the coverage and this effect was most pronounced for haplotypes occurring at low frequencies (Figure 2A), as especially for those haplotypes an increase in coverage has a relatively larger effect on the ability to distinguish a true polymorphism from a sequencing error. Figure 2A also shows that even at low coverage (50-fold), haplotypes were reconstructed with an average reliability of 70%. For the same coverage the performance improves with a decrease in the complexity of the pool (less polymorphisms and less haplotypes), with a maximum of 92% in average reliability observed for the least complex problem (100 polymorphisms, 2 haplotypes, at the highest coverage of 500-fold) (Supplementary Figure S4).

Supplementary Figure S4 shows for the different simulated set ups, the degree to which EVORhA could correctly estimate the true haplotype frequencies in the population (expressed by the MAE). The ability to estimate true frequencies seems largely independent of the number of haplotypes in the population or the number of polymorphisms. The latter is to be expected as the total number of polymorphisms in the simulation is sparse anyhow and does confer little information to the final frequency estimation. Figure 2B shows how the true frequency estimation is largely affected by the coverage: when the coverage is low (50-fold), the average error rate on estimating the true frequency of the haplotypes is around 10%. This is understandable given that at low coverage the sampling that produces the reads is more prone to random effects.

### Comparison of EVORhA with state-of-the-art haplotype reconstruction

Because our method builds for its initial step on concepts that were first described in the context of viral haplotype reconstruction, we compared our tool with state-of-the-art viral haplotype reconstruction tools. As representatives of read-graph based tools we used ShoRAH (11) and QuasiRecomb (16), both widely used for viral haplotype reconstruction. In addition, we used PredictHaplo (15) as a representative of probabilistic haplotype reconstruction methods.

As none of the above mentioned viral haplotype reconstruction tools (ShoRAH, QuasiRecomb and PredictHaplo) was able to run in the bacterial setting, we compared our method in a setting more appropriate for these state-of-the-art tools (reconstruction of viral sized haplotypes in the presence of a large number of polymorphisms). Hereto, we designed a simulation experiment, mimicking the data resulting from the population sequencing of a small region obtained with either a relatively short or long read sequencing technology (respectively mimicking Illumina and Roche 454 reads). Simulated populations differed from each other in the number of haplotypes (ranging from 2 to 4), the used sequence coverage (50-, 100-, 200- and 500-fold) and the number of polymorphisms in the population (7, 10, 20 and 50 sites). For each experimental setup 100 different data sets were simulated and performances of respectively EVORhA, ShoRAH and QuasiRecomb were assessed as outlined above and in the material and methods.

**Figure 3.**
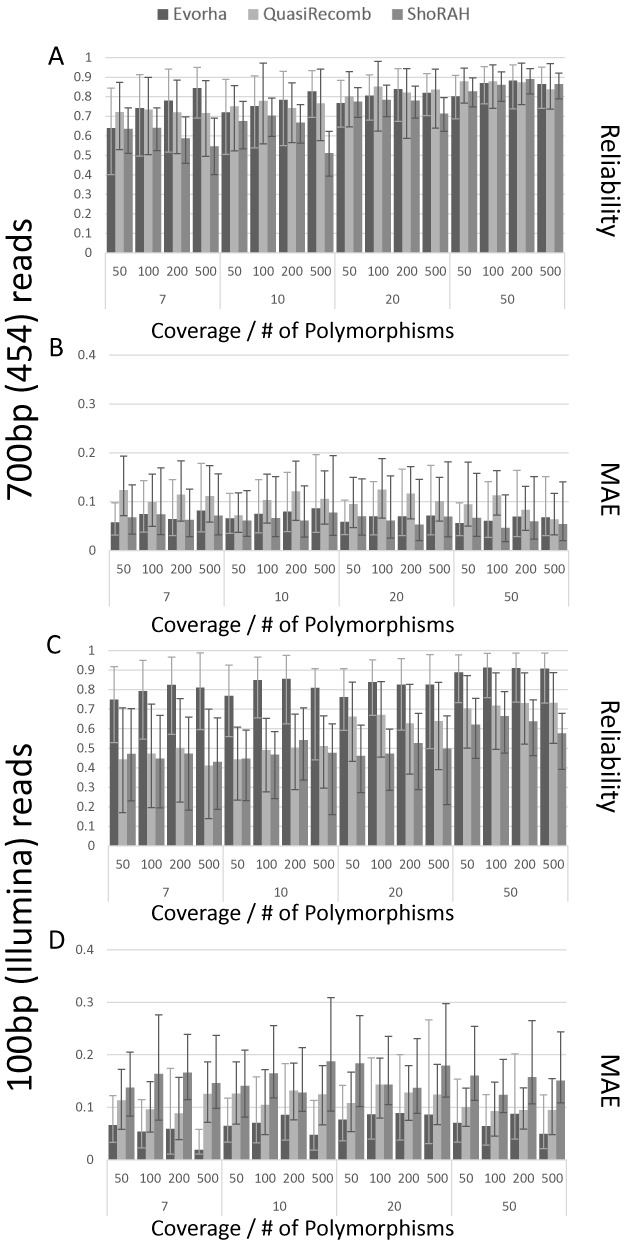
Performance comparison of EVORhA, ShoRAH and QuasiRecomb on simulated data. (**A**) Comparison of the reconstruction reliability using long read sequencing (700 bp), hereby collapsing the results obtained for simulations with a different number of haplotypes. Data sets were obtained by simulating long read sequencing. The X-axis displays the different combinations of coverage (respectively 50, 100, 200, 500) and number of polymorphisms in the population (respectively 7, 10, 20, 50) used for each experimental set up. The Y-axis shows the average reliability of the reconstruction. Bars indicate the performance per method. Reliability values are obtained by averaging the reliability scores of all haplotypes resulting from simulations obtained with the same coverage and same number of polymorphic sites, irrespective of the number of haplotypes. Error bars indicate the 90% confidence interval of the reconstruction. (**B**) Comparison of the frequency estimation of the haplotypes using long read sequencing. Experimental set up and legend as in panel (A) except for the Y-axis which displays the MAE of the frequency estimation for all haplotypes resulting from simulations obtained with the same coverage and same number of polymorphic sites, irrespective of the number of haplotypes. Error bars indicate the MAE 90% confidence interval. (**C**) Comparison of the reconstruction reliability using short read sequencing (100 bp). Legend and experimental set up as in panel (A), but displaying results obtained on data simulating short reads. (**D**) Comparison of the reconstruction reliability using short read sequencing. Legend and experimental set up as in panel (B), but displaying results obtained on data simulating short reads.

Figure 3 shows that in general, and irrespective of the read length used for sequencing, using an increased sequencing coverage and having intrinsically more polymorphic sites in the population positively influences the performance of all methods, mainly in terms of reliability i.e. correctly reconstructing the true haplotypes in the population. Only for ShoRAH the reconstruction reliability seems to decrease with the coverage in case haplotypes were obtained from populations with few polymorphisms (<10).

For relatively long reads (700 bp), EVORhA reaches performances similar to those of QuasiRecomb and ShoRAH: in the tested setting the reconstruction reliability obtained with QuasiRecomb was slightly higher than the one obtained with EVORhA, but this came at the expense of QuasiRecomb having a relatively lower performance in terms of the frequency estimation (relatively higher MAE) than EVORhA.

For shorter reads (100 bp) EVORhA consistently outperforms QuasiRecomb and ShoRAH, both in terms of having a higher reconstruction reliability and having a better frequency estimation.

### Haplotype reconstruction to reconstruct evolutionary trajectories

**Figure 4.**
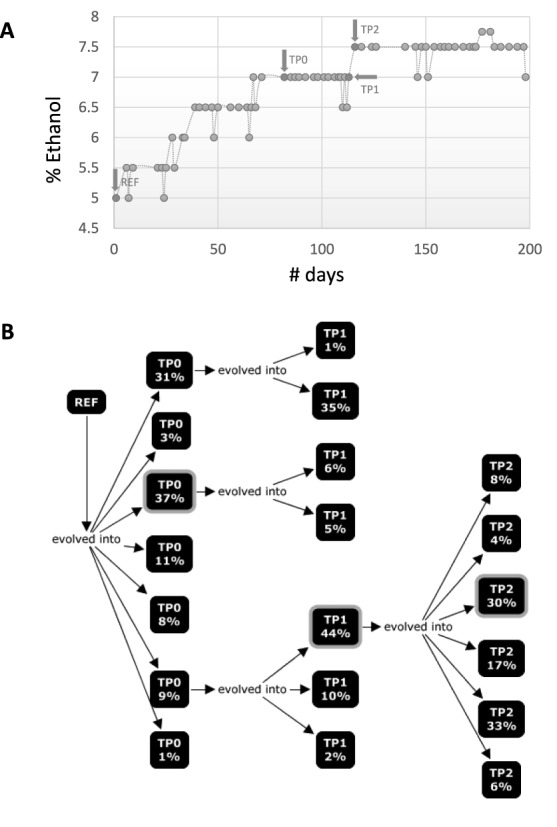
Haplotype reconstruction to infer evolutionary trajectories. (**A**) shows the phenotypic trajectory of a population during an evolution experiment in which *E. coli* strains were subjected to increasing ethanol concentrations. The measured focal phenotype is the ethanol tolerance of the population (i.e. the % of ethanol at which growth still occurs). Arrows indicate the time points at which population samples were taken that were subjected to sequencing and haplotype reconstruction. Y-axis indicates the % of Ethanol to which the population was subjected. (**B**) concept map illustrating the evolutionary relations between the haplotypes reconstructed from each of sampled time points described in panel (A). Ref indicates the unevolved parental strain of which the genomic sequence was used as a reference. TP0, TP1 and TP2 represent the 3 time points at which population samples were taken (see panel (A)), i.e. TP0 is Time Point Zero (0). Each square corresponds to a different reconstructed haplotype and ‘%’ indicates the frequency at which this haplotype was estimated to occur in the population. Arrows indicate the phylogenetic relatedness between the reconstructed haplotypes (or ancestry). Indicated with a lighter gray border are the reconstructed haplotypes that best match the individual clones, sampled at each time point.

To test EVORhA in a real setting, we reconstructed haplotypes from pooled sequencing data of population samples, taken during an evolution experiment. In this experiment a lab *E. coli* strain was subjected to high ethanol concentrations and growth in the presence of ethanol, referred to as ethanol tolerance was estimated as a focal phenotype. The trajectory of the population phenotype clearly showed that after about 100 days, the cell's ethanol tolerance steadily increased from 7% to 7.5% after which a plateau was reached (Figure 4A). To evaluate the evolutionary trajectories of the haplotypes during this switch in the population phenotype, samples were taken at three consecutive time points: at T0, the beginning of a 40 days stationary phase when no increased tolerance against ethanol was observed yet, at T1 right before the phenotypic switch and at T2 the focal end point after which no further increase in ethanol tolerance was observed (Figure 4A). Population samples were subjected to Illumina pooled sequencing and applying EVORhA to the data obtained for each of the pooled samples allowed reconstructing per population its composition i.e. the different haplotypes that were present and the frequencies at which they were present in the respective populations. As haplotypes present in consecutive time points are related to each other through their common ancestry, the phylogenetic relations between the reconstructed haplotypes was inferred, the evolutionary history is represented by means of a concept map using CmapTools (23) (Figure 4B).

To verify the correctness of the haplotype reconstruction, we sampled and sequenced one clone per time point and determined their polymorphisms. Per time point each sampled clone was assigned to its ‘best matching reconstructed haplotype’ based on a minimal number of inconsistencies between polymorphisms of the sampled clone and polymorphisms present in the inferred haplotypes. At TP0, the genome of the sampled clone contained 296 polymorphisms that were still rising to fixation in the population. 295 of these polymorphisms could uniquely be assigned to one of the reconstructed haplotypes (i.e. with a reliability of 99.7%). At TP1 the sampled clone contained 152 polymorphisms rising to fixation in the population, of which 151 could uniquely be assigned to a reconstructed haplotype (with 99.3% reliability). At TP2, the sequenced clone contained 122 polymorphisms still rising to fixation in the population of which 111 could uniquely be assigned (with 91% reliability). The haplotypes best matching the genomic sequences of the sampled clones are indicated in Figure 4B.

Although the sampled clones did most often correspond to the haplotypes that were high in frequency at the time of sampling, reconstructing the evolutionary trajectory of the focal end point clone would not have been possible using the sequence information from the sampled clones only. This because, although phenotypically the population undergoes a clear increase in ethanol tolerance (Figure 4A), it remains at each single time point largely heterogeneous (Figure 4B). The two haplotypes dominating at TP0, before the sweep with a frequency of respectively 31% and 37%, of which one was sampled as an individual clone, seem to have been dead ends and therefore were not ancestral. In contrast, a minor haplotype rapidly increasing in frequency between TP0 and TP1 (from 9% to 44%) took over the population and eventually gave rise to all haplotypes at TP2, the focal end point.

Note that in Figure 4B, frequencies inferred at a single time point do not sum to one. This because, we intentionally choose to only perform a partial haplotype reconstruction in case the genome-wide reconstruction was still ambiguous (if a haplotype set occurs at a low frequency and has no conflicts with more than one haplotypes occurring at higher frequency, we have insufficient information to decide to which genome-wide haplotype the haplotype set at low frequency belongs).

### Haplotype reconstruction to identify mixed infections

Currently, tracing the origin of an infectious disease during an outbreak is based on determining the genetic similarity between individual strains sampled from different infected entities (individuals), hereby assuming that the contaminating population isolated from each entity is largely homogenous. However, in case of mixed infections such approach might fail (22) unless the different contaminators within one individual can be disentangled.

**Figure 5.**
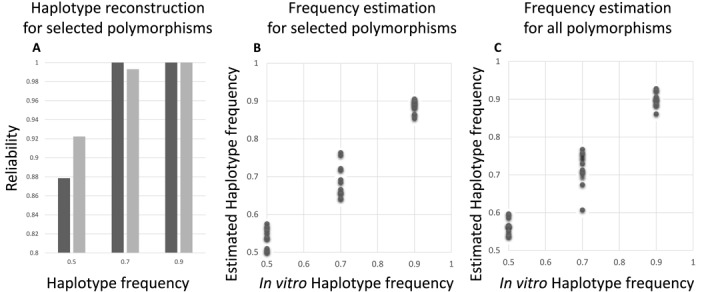
Haplotype reconstruction to identify mixed infections. (**A**) Reliability of the haplotype reconstruction of the mixed infection set up when using a selection of polymorphisms. The X-axis contains the three different *in vitro* proportions at which the mixed infection set ups were generated (50–50%, 30–70% and 10–90%). For each of the three proportions, 12 mixtures were obtained by mixing two different clonal DNA samples according to the indicated frequencies. The Y-axis indicates the average reliability of the two reconstructed haplotypes compared to the known haplotypes at the polymorphic sites. (**B** and **C**) Correctness of the frequency estimation in the mixed infection set up. For panels (B) and (C) the X-axis represents the known frequency of this haplotype in the mixture, whereas the Y-axis represents the estimated frequencies of the most frequent haplotype in each combination (the least frequent haplotype is not displayed as it would have a frequency of 1—the shown frequency). (B) Correctness of the frequency estimation based on a haplotype reconstructing using the 151 selected polymorphisms only (RMSE = 0.037, MAE = 0.030). (C) Correctness of the frequency estimation based on a haplotype reconstructing using all polymorphisms (RMSE = 0.047, MAE = 0.038).

To assess the applicability of EVORhA in identifying mixed infections, we used the benchmark data generated by Eyre et al. (22). These authors mimicked *in vitro* 36 mixed *Clostridium difficile* infections by pairwisely combining in different proportions (50%/50%, 70%/30% and 90%/10%) DNA extracted from single clones. We reconstructed genome-wide haplotypes from the Illumina based sequence data of these mixed samples. To this end, we used EVORhA either in combination with ‘all’ polymorphisms detected in the population or as an alternative, and consistent with the original approach described in Eyre et al., in combination with a preselected set of polymorphisms a priori known to be discriminative for the haplotypes in the mixture (22). Also here, the reliability of the haplotype reconstruction was assessed by comparing the reconstructed genomes with the ones known to be present in the mixtures. The correctness of the inferred haplotype frequencies was assessed by the RMSE. Results for the reconstruction are displayed in Figure 5.

For both approaches, it is clear that reconstructing haplotypes from a mixed infection improves if the haplotypes in the mixture occur at different frequencies (such as observed at 90%/10% or 70%/30%). This is reflected by the high reliabilities (Figure 5A) and good frequency estimates (Figure 5B and C) obtained at these frequency ratios in the mixture. This is not unexpected as in this bacterial setting (low mutation frequency) the haplotype reconstruction largely relies on the frequency information: the more the frequencies differ between the haplotypes in the mixture, the more discriminative this feature is in assigning polymorphisms to correct haplotypes. Although in 50%/50% mixtures many ambiguous assignments are expected to occur, resulting in the lowering of the reconstruction reliability, it is remarkable that EVORhA is still able to reconstruct the haplotypes relatively well in the 50%/50% mixture (a reliability of at least 85% which is significantly higher than what would be expected from randomly combining contigs occurring at 50% into two haplotypes). At the given sequencing coverage, the mixture model underlying EVORhA seems to have a rather high resolution (allowing to separate a haplotype occurring at 53% from a haplotype occurring at 47%). Although this deviation from 50% is now penalized in the RMSE, which assumes that in the *in vitro* mixed sample the haplotypes truly occur at 50%/50%, experimental and sampling biases might have resulted in the inferred small deviations of this intended 50%/50%. Despite being very small, these frequency deviations can still be captured by the haplotype reconstruction, resulting in a correct reconstruction.

## DISCUSSION

In this work we present EVORhA, a method for reconstructing haplotypes from deep sequencing data of clonal populations that have a relatively low mutation rate, such as bacteria.

Haplotype reconstruction in general is complicated because polymorphisms of infrequent haplotypes are difficult to distinguish from sequencing errors. The solution to this problem, referred to as local haplotype reconstruction has been proposed in the context of viral haplotype reconstruction and relies on simultaneously, rather than sequentially identifying sequencing errors and reconstructing haplotypes (11–16). EVORhA uses a local haplotype reconstruction based on similar principles, but in addition exploits the information contained in a BLOSUM matrix to better distinguish true polymorphisms from likely sequencing errors.

In contrast to viral reconstruction, however, in our bacterial setting read lengths are short compared to the average distance between polymorphic sites. This prevents us from using viral haplotype reconstruction approaches to infer bacterial haplotypes, because in order to extend locally inferred haplotypes into more global ones, all viral methods rely on the presence of a sufficient number of segregating sites to reconstruct haplotypes from phasing information (10).

Key to our method, therefore, is the use of the frequency ratios of the inferred haplotypes, not only to improve the extension of haplotypes for which the non-empty overlap between flanking windows results in an ambiguous phasing (i.e. the window extension) (17), but also to further link distant phased regions that have no sequence overlap at all (i.e. during the genome-wide reconstruction step).

As was shown on the simulated data, as soon as read lengths and/or mutation rates become restrictive for state-of-the-art methods, the additional frequency information, mainly through the genome-wide reconstruction step allows EVORhA to still reliably reconstruct haplotypes.

This frequency-based genome-wide reconstruction is also the key enabling step to resolve the bacterial haplotypes in the real data applications. This step, based on using a mixture model assumes that locally extended haplotypes observed at similar frequencies are likely to belong to the same global haplotype. This assumption gets violated however, if two haplotypes occur at similar frequencies, in which case the haplotype reconstruction might result in hybrids. However, our results on the mixed infection data set showed that even marginal frequency deviations between haplotypes allow the mixture model to resolve these haplotypes with high accuracy, provided the sequencing coverage of the sample is sufficiently high.

As was shown in the results, sequencing coverage highly impacts the reconstruction performance of EVORhA: at first indirectly because it affects the correctness of the reference-based assembly which is used as input. More directly because a too low coverage complicates distinguishing sequencing errors from true polymorphisms. In addition, the coverage determines the maximum number of haplotypes that can be detected. This is mainly because the standard deviation used when inferring the mixture model is dependent on the coverage, i.e. a lower coverage implies larger standard deviations of the Gaussian distributions of the mixture model which may cause haplotypes occurring at similar frequencies to become confounded.

Conclusively, EVORhA, by enabling bacterial haplotype reconstruction opens a whole new area of applications for bacterial population sequencing (or metagenome sequencing). As was illustrated by the real data examples, bacterial haplotype reconstruction can aid in resolving mixed infections or in reconstructing the dynamics of evolving clonal populations. In addition, it can potentially be useful to further resolve genomes from reads with similar sequence composition in bacterial metagenomics data sets of which the complexity has been reduced with binning approaches (24–26).

Haplotype reconstruction thus provides a quick view on the composition of a mixed sample and allows pinpointing haplotypes with interesting characteristics that can be further focused on by downstream molecular analysis.

## SUPPLEMENTARY DATA

Supplementary Data are available at NAR Online.

SUPPLEMENTARY DATA
