# Supplementary material for: Frequency-based haplotype reconstruction from deep sequencing data of bacterial populations
Source: Nucleic Acids Res. 2015 May 18;43(16):e105. doi: 10.1093/nar/gkv478 (PMC4652744; doi:10.1093/nar/gkv478)
Supplement: SUPPLEMENTARY DATA [file supp_43_16_e105__index.html]

Frequency-based haplotype reconstruction from deep sequencing data of bacterial populations — Frequency-based haplotype reconstruction from deep sequencing data of bacterial populations — SUPPLEMENTARY DATA 

# Frequency-based haplotype reconstruction from deep sequencing data of bacterial populations

## SUPPLEMENTARY DATA

- SUPPLEMENTARY DATA
- SUPPLEMENTARY DATA
- SUPPLEMENTARY DATA
- SUPPLEMENTARY DATA
- SUPPLEMENTARY DATA
