# Supplementary material for: Frequency-based haplotype reconstruction from deep sequencing data of bacterial populations
Source: Nucleic Acids Res. 2015 May 18;43(16):e105. doi: 10.1093/nar/gkv478 (PMC4652744; doi:10.1093/nar/gkv478)
Supplement: SUPPLEMENTARY DATA [file supp_gkv478_nar-00532-met-n-2015-File007.docx]

**Figure 1S**. **Inferring template haplotypes and performing error correction at the local scale**. The example population consists of three haplotypes (**A**: *Green* at 55%, *Blue* at 30% and *Purple* at 15%). Reads obtained from sequencing this population were mapped to a reference genomic region (Black), allowing the detection of polymorphic sites. To differentiate true variants from sequencing errors in a window (**B**), a set of template haplotypes is defined from those reads that fully cover the window (indicated in respectively lighter *green*, *blue*, *purple* and *grey)*. For each template haplotype a *support* (**C**) is calculated using 1) the reads that fully overlap with the window (i.e. *base support*, hereby assuming a perfect base call accuracy for simplification purposes, i.e. $w\left( r \right)=1$) , but also using 2) all remaining reads that partially overlap with the window and that are consistent with the template haplotypes (i.e. *additional support*). These partially overlapping reads give an additional support to each template haplotype with which they are consistent, proportional to the *base support* of each template haplotype. In the example represented here, the three yellow reads match to the *green*, *blue* and *grey* haplotypes (as they do not contain a mutation in SNP1). Each yellow read gives additional support to all consistent template haplotypes, taking into account the *base support* of the matching haplotypes e.g. 8 is the base support of the green haplotype, 4 is the base support of the blue and 1 of the grey, see figure insets **B** and **C**. The sum of the haplotypes base supports matching the yellow reads is 13. Therefore, the contribution of each yellow read to the additional support of the matching haplotypes is calculated as follows: 8/13 for green, 4/13 for blue and 1/13 for grey. Only template haplotypes with a sufficient total support will be maintained for further analysis, hereby assuming that erroneous template haplotypes that are the result of a sequencing error will not have sufficient total support. In the example, the grey template ends up with a total support of 1.42 (1 base support + 3 yellow reads that each gives 1/13 additional support + 2 pink reads that each gives 1/11 additional support) while the other haplotypes obtain a much larger total support: 3.96 for purple (2 base support + 1 partially overlapping read only consistent to the purple template + 3 magenta reads with 2/11 additional support each + 2 pink reads with 2/11 additional support), 9.93 for blue (4 base support + 5 additional support from reads with partial overlap consistent only to the blue template + 3 yellow reads with 4/13 additional support each) and 17.70 for green (8 base support + 4 partially overlapping reads consistent only to this template + 3 yellow reads with 8/13 additional support each + 3 magenta reads with 8/10 additional support each + 2 pink reads with 8/11 additional support each).

**Figure 2S.** **Window extension procedure**. In the example four windows contain reads from the sequencing of a population of three haplotypes (*green*, *blue* and *purple*). These windows can be extended because they have polymorphisms in their read overlap. EVORhA choses a *seed* window to start the window extension (say, Window 2). Window 2 is extended with its flanking windows (window 1 and window 3) starting by the one on the left. Groups are declared by combining template haplotypes from both windows that share the same unique combination of polymorphisms in the overlap region of the windows. In this case **(B)** there are two groups: template haplotypes belonging to group 1 share the SNP in their overlap region, whereas those of group 2 share “not having the SNP”. The goal of the extension is to find within a group the best concatenation of template haplotypes from respectively the first and second window to generate an extended haplotype, where ‘best’ is defined in terms of matching frequencies and shared polymorphisms. **(A)** A toy example of an extension is given below: we start by joining window 1 and 2 **(B)**, in which two groups can be distinguished. The extension is performed for each group separately. This is an example of two straightforward extensions as group 1 only contains exactly one template haplotype in either window, whereas group 2 only contains one haplotype in window 1. For both groups the extension consists of concatenating the template haplotypes of the flanking windows. The extended haplotypes belong to an artificial window referred to as Concatenated Window 1 **(C)**, which will be further extended with window 3 **(D)**. Again, the template haplotypes are separated in two groups, a group containing the template haplotypes that share the SNP (group 1) and those without the SNP (group 2) in their overlap region. The extension of group 1 is again straightforward and a concatenation is sufficient. For group 2 the extension is ambiguous **(E)** as the concatenation can result either in the true haplotypes or a chimera. To solve this, an Expectation Maximization algorithm is used to find which concatenation is the most likely based on the frequencies of the template haplotypes within a group. So, we assume that a template haplotype in a window will have a more similar frequency with its true counterpart in the flanking window than with any other template haplotype in the same flanking window. In the next extension, when extending using window 4, we can only divide the haplotypes into a high complexity extendable group where many more combinations are possible. The window overlap information here becomes null as all haplotypes share the same mutations. The responsibility of the EM is to find the most likely way to concatenate the haplotypes based on the haplotype frequencies in the group.

**Figure 3S**. **Frequency analysis**. Four genome-wide haplotypes were simulated, occurring at a frequency of respectively 10%, 20%, 30% and 40% in a given population. Pooled sequence data were simulated and the frequency with which ‘extended haplotypes’ were detected in each concatenated window was recorded. The histogram displays the number of extended haplotypes (Y-axis) that occur at a given frequency (X-axis). This histogram shows how several extended haplotypes, here referred to as haplotype sets occur at approximately the same frequency. The simulation shows that the distribution of the frequency at which extended haplotypes sets occur can be modelled by six independent Gaussian distributions. Four of these Gaussians correspond to haplotype sets containing polymorphisms unique to each of the respective simulated genome-wide haplotypes (*blue*, *purple*, *yellow* and *cyan*), whereas the two Gaussians observed at higher frequencies correspond to haplotype sets containing polymorphisms shared by several genome-wide haplotypes (*orange* and *red*). The goal of the frequency analysis is to determine a mixture model of Gaussian distributions that optimally describes the observed frequencies at which the haplotype sets occur. Extended haplotypes belonging to the same set likely belong to the same ‘genome-wide haplotype’ provided they do not contain any conflicting polymorphisms at the same polymorphic site. The haplotype sets are subsequently used to infer genome-wide haplotypes, hereby assuming that for each considered haplotype set a combination of haplotype sets might exist, occurring at a frequency lower than the frequency of the considered haplotype set and for which the sum of these respective frequencies equals the frequency of the haplotype set under consideration e.g. the *orange* haplotype set frequency is the sum of the *cyan* and *purple* frequencies.

**Figure 4S. Reliability of haplotype reconstruction by EVORhA on simulated data: effect of the complexity of the pool.** The X-axis displays the different number of haplotypes in the population (from 2 to 7) and the number of polymorphisms (respectively 100, 1000, 2500) used for each simulated set up. The degree to which the simulated haplotypes was correctly reconstructed was assessed by the average reliability. The correctness of the frequency estimation of the reconstructed haplotypes was assessed by the Mean Absolute Error (MAE). Panel **A**, **B** and **C)** Reliability of the haplotype reconstruction for 50, 200 and 500 fold coverage, respectively. Y-axis: average reliability; values represent the average of the reliability values obtained for all haplotypes recovered from the 100 runs obtained per experimental set up (similar coverage, similar number of polymorphisms, similar number of haplotypes in the population). Error bars indicate the 90% confidence interval of the reconstruction. Panel **D**, **E** and **F)** Y-axis: Mean Absolute Error (MAE) of the frequency estimation for 50, 200 and 500 fold coverage, respectively. Values represent the Mean Absolute Error (MAE) for all haplotypes recovered from the 100 runs obtained per experimental set up (similar coverage, similar number of polymorphisms, similar number of haplotypes in the population). Error bars indicate the MAE 90% confidence interval.
