# Supplementary figures and images for: Frequency-based haplotype reconstruction from deep sequencing data of bacterial populations
Source: Nucleic Acids Res. 2015 May 18;43(16):e105. doi: 10.1093/nar/gkv478 (PMC4652744; doi:10.1093/nar/gkv478)

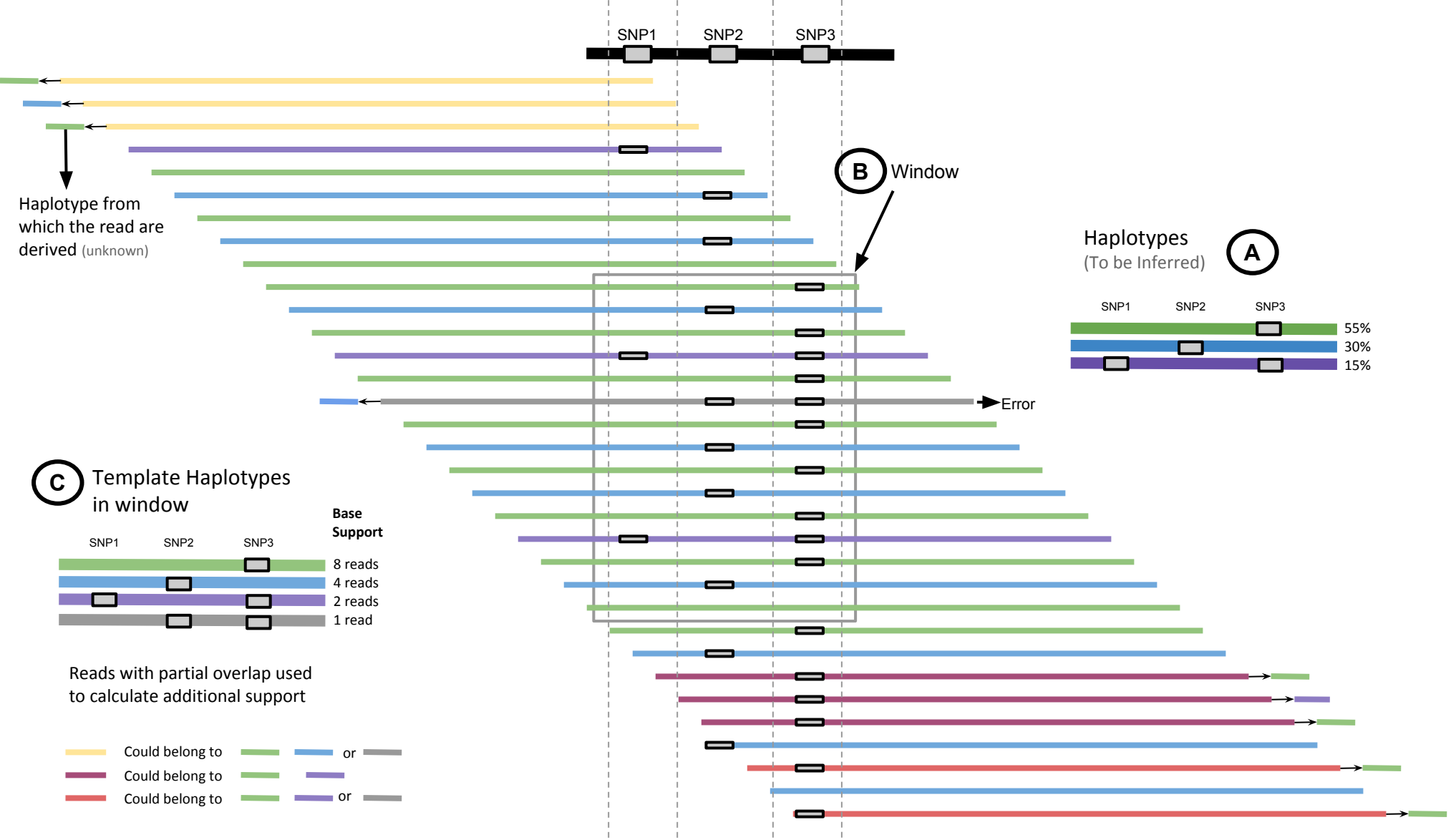

Supplement: SUPPLEMENTARY DATA [file supp_gkv478_nar-00532-met-n-2015-File008.pdf]

**A**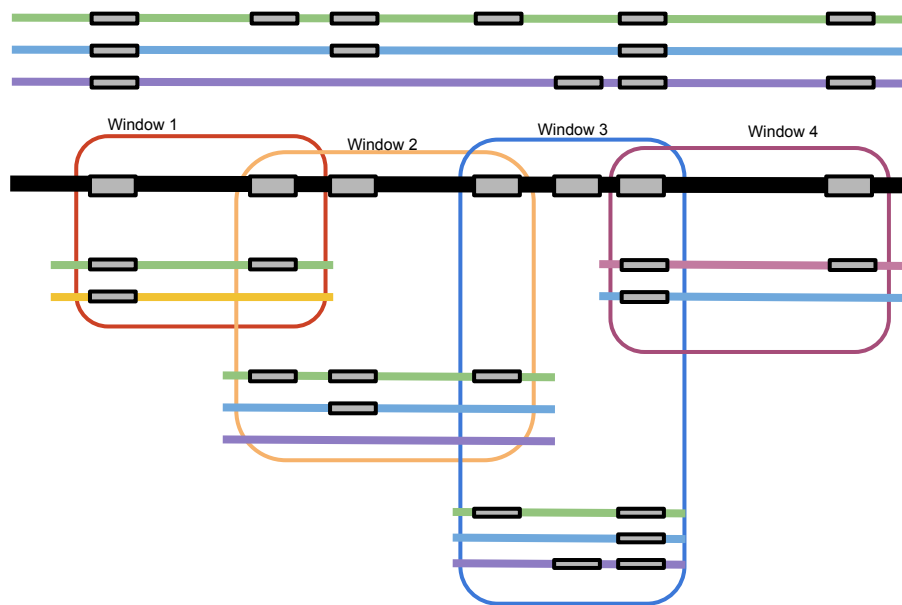**B**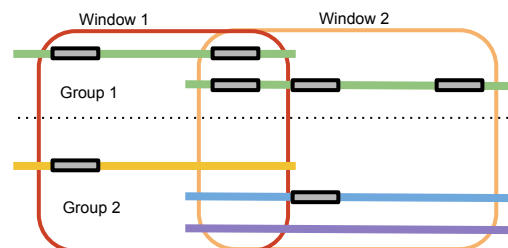**C**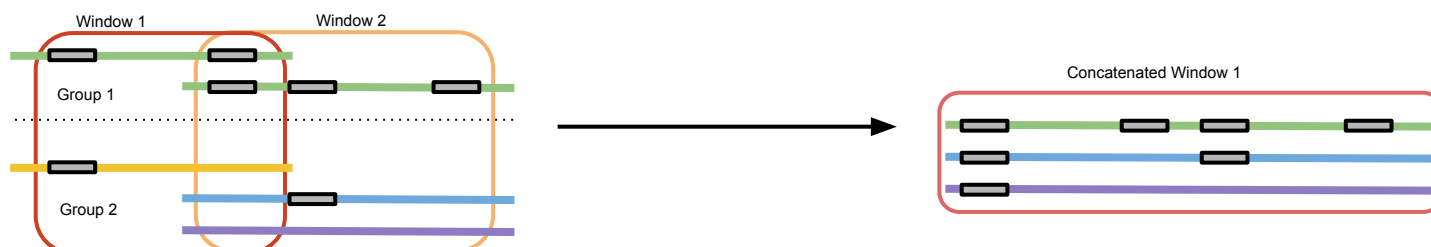**D**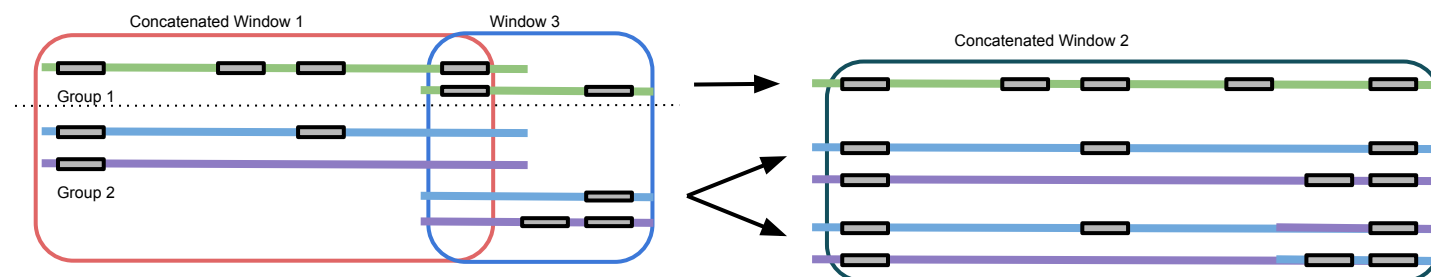**E**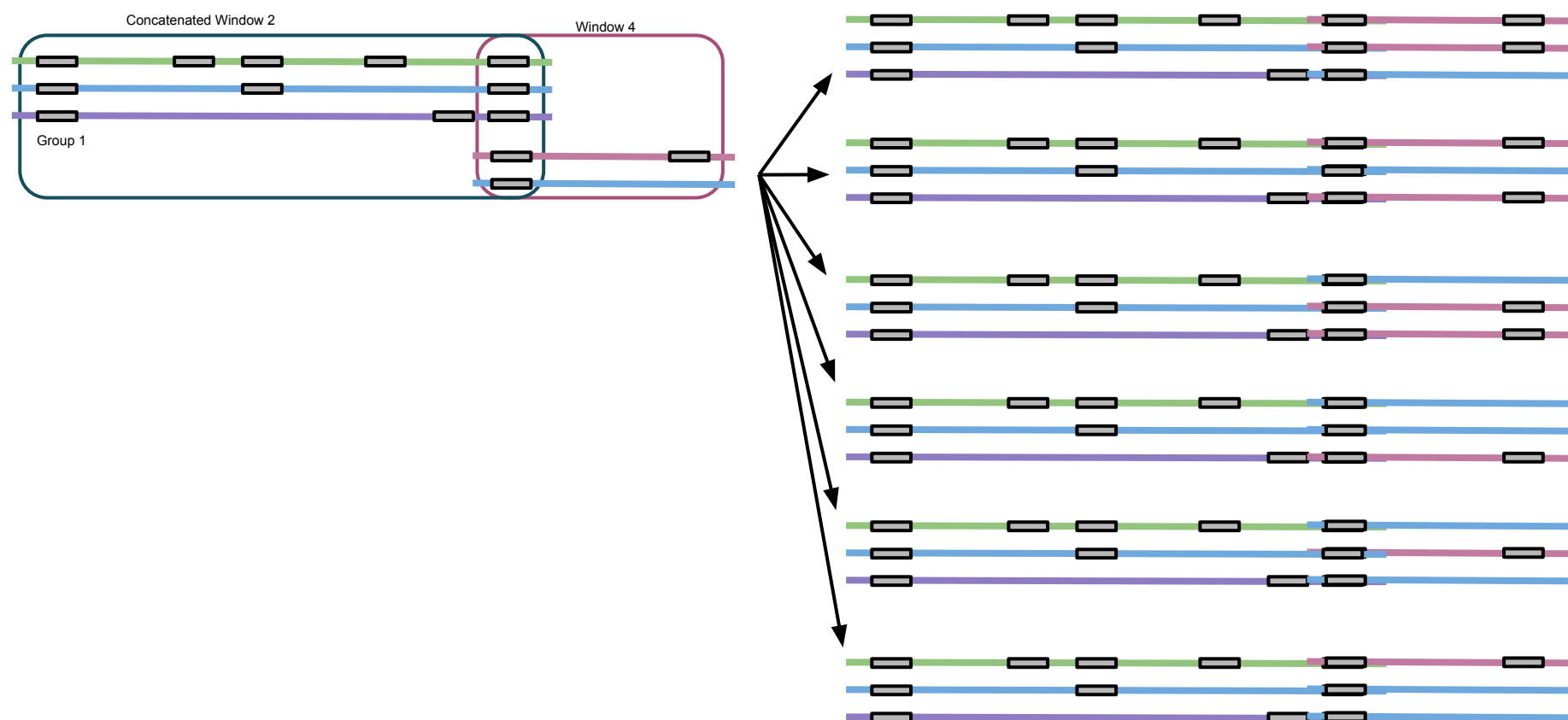

Supplement: SUPPLEMENTARY DATA [file supp_gkv478_nar-00532-met-n-2015-File009.pdf]

Coverage 50

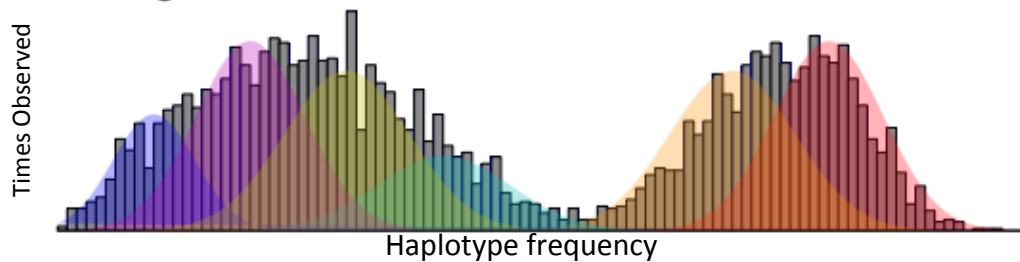

Coverage 100

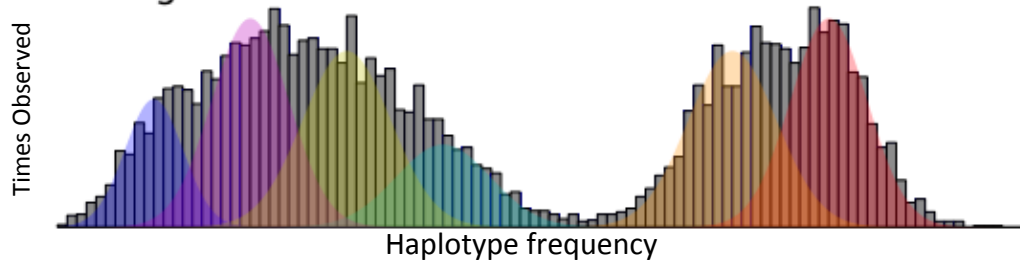

Coverage 500

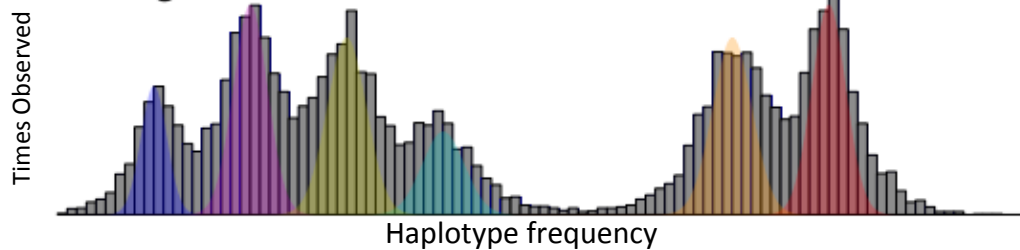

Supplement: SUPPLEMENTARY DATA [file supp_gkv478_nar-00532-met-n-2015-File010.pdf]

Reliability

MAE

50 fold

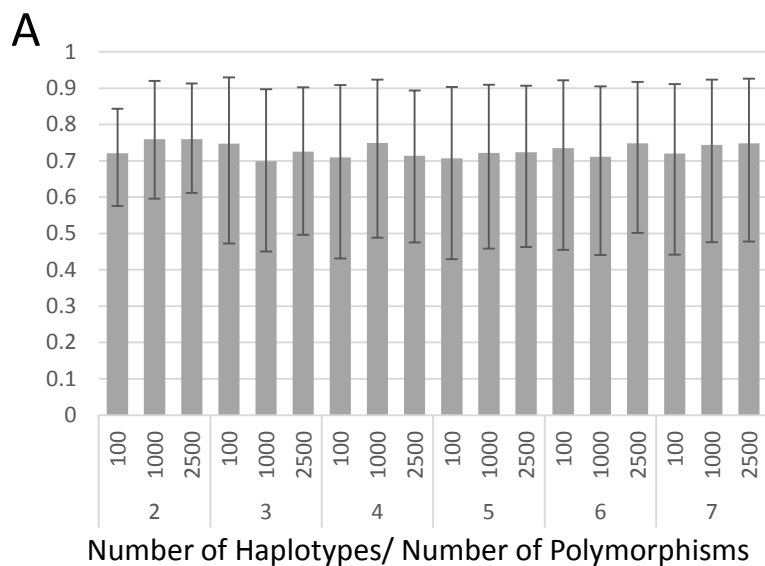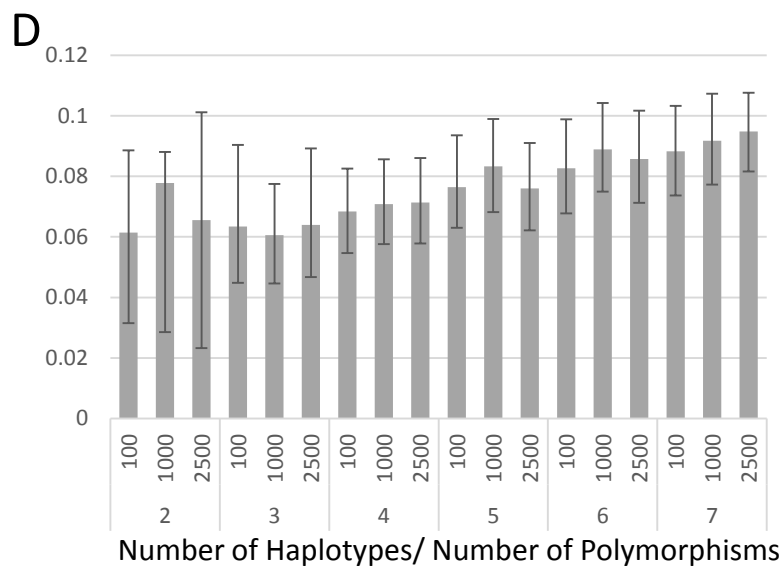

200 fold

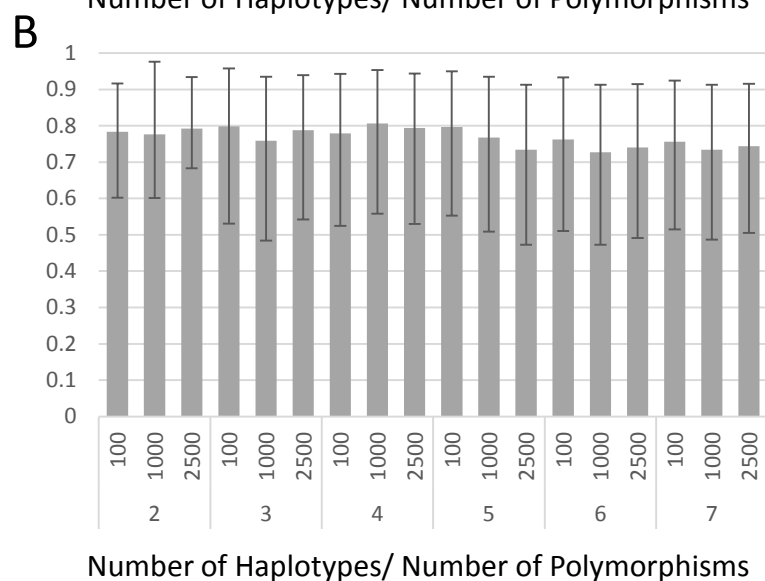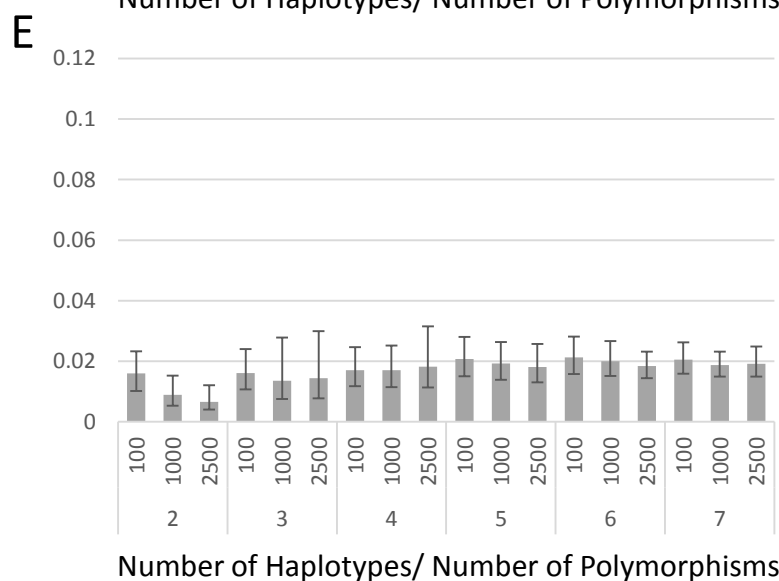

500 fold

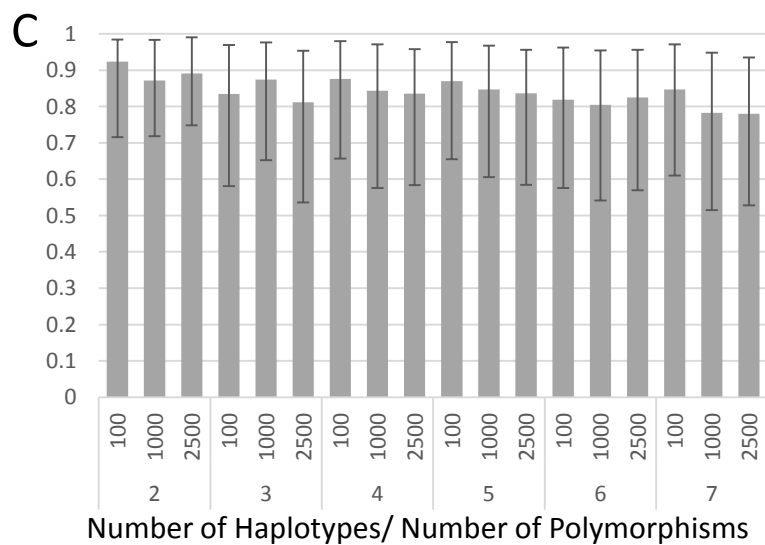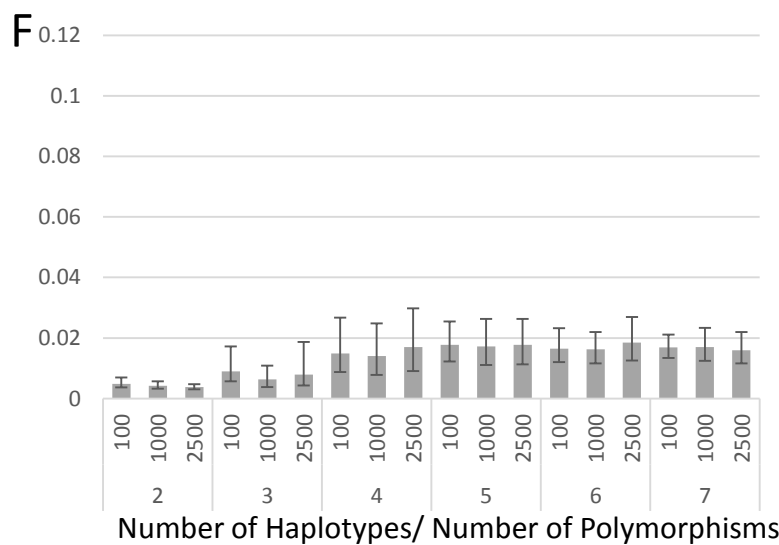

Supplement: SUPPLEMENTARY DATA [file supp_gkv478_nar-00532-met-n-2015-File011.pdf]
